# In silico identification of drug target pathways in breast cancer subtypes using pathway cross-talk inhibition

**DOI:** 10.1186/s12967-018-1535-2

**Published:** 2018-06-05

**Authors:** Claudia Cava, Gloria Bertoli, Isabella Castiglioni

**Affiliations:** Institute of Molecular Bioimaging and Physiology, National Research Council (IBFM-CNR), Via F.Cervi 93, Segrate, 20090 Milan, Italy

**Keywords:** Monte Carlo cross-validation, Pathway cross-talk inhibition, Breast cancer, Drugs, Classification, Subtypes

## Abstract

**Background:**

Despite great development in genome and proteome high-throughput methods, treatment failure is a critical point in the management of most solid cancers, including breast cancer (BC). Multiple alternative mechanisms upon drug treatment are involved to offset therapeutic effects, eventually causing drug resistance or treatment failure.

**Methods:**

Here, we optimized a computational method to discover novel drug target pathways in cancer subtypes using pathway cross-talk inhibition (PCI). The in silico method is based on the detection and quantification of the pathway cross-talk for distinct cancer subtypes. From a BC data set of The Cancer Genome Atlas, we have identified different networks of cross-talking pathways for different BC subtypes, validated using an independent BC dataset from Gene Expression Omnibus. Then, we predicted in silico the effects of new or approved drugs on different BC subtypes by silencing individual or combined subtype-derived pathways with the aim to find new potential drugs or more effective synergistic combinations of drugs.

**Results:**

Overall, we identified a set of new potential drug target pathways for distinct BC subtypes on which therapeutic agents could synergically act showing antitumour effects and impacting on cross-talk inhibition.

**Conclusions:**

We believe that in silico methods based on PCI could offer valuable approaches to identifying more tailored and effective treatments in particular in heterogeneous cancer diseases.

**Electronic supplementary material:**

The online version of this article (10.1186/s12967-018-1535-2) contains supplementary material, which is available to authorized users.

## Background

Breast cancer (BC), the most invasive cancer in women worldwide, is a heterogeneous disease, characterized by different subtypes that lead to different clinical prognosis and responses to treatments [1].

The advent of genome-wide technologies has made possible the generation of new hypotheses about the role of genomics in the efficiency of drugs developed for cancer and the event of adverse responses to cancer therapy.

In this context, several studies examined the effects of drugs considering protein network approaches [2]. In particular, the analysis of network models revealed that the partial inhibition of a small number of proteins belonging to a network has a higher impact on the disease than the complete inhibition of a single protein: indeed, drugs that have only one target (single hit) often do not affect complex system networks of proteins in an effective way [2]. Moreover, experimental studies have shown that cancer cells are able to resist to drug treatments by creating and establishing news interactions in order to have an alternative signaling [3]. In network approaches, the effect of a drug treatment on some proteins, represented by the nodes of a network, is amplified by the interactions of these proteins with other proteins in the networks, being these connections represented by edges [2]. However, notwithstanding useful to assess the drug effects on proteins, these approaches have not still impacted in increasing the number of efficient drugs or in suggesting new potential targets for cancer treatment.

Hence, two critical points for drug discovery tools are: (i) to inhibit not single but multiple targets at the same time, and (ii) to prevent the formation of new interactions that could lead to phenomena of resistance or inefficacy of the drug.

Moreover, these two critical conditions can be further complicated by the interactions between pathways (pathway cross-talk) that can modify the effect of drugs.

For instance, in some cancer cells, rapamycin-like drugs inhibit mTORC1 complex but at the same time indirectly activate phosphatidylinositol 3-kinase (PI3-kinase) and AKT, mitigating the inhibitor effect of drugs [3]. Similarly, in triple negative BC the inhibition of AKT as a consequence of a drug is indirectly damped by the activation of receptor tyrosine kinases (RTKs), reducing the efficacy of the drug [4]. Moreover, pathway cross-talk, i.e. the one existing between EGFR and HER2, is a possible evasive way for the cell to develop resistance to HER family receptor inhibitors [5]. These examples illustrate how drugs targeting an individual protein or pathway may not yield to the expected therapeutic effect due to activation of alternative pathways that avoid the barrier inflicted by the cancer drug.

Therefore, understanding how and where the pathway cross-talk can be inhibited by drug treatments during a disease process ideally could lead to more effective therapies, reducing the problem of drug resistance.

Once a map of pathway cross-talk specific of a disease is known, a potential solution hindering the formation of alternative signaling pathways created in response to therapy could be the administration of drugs able to act on both direct and indirect targets (generated by the inhibition of direct targets).

However, on this potential solution, the current opinions are conflicting. Indeed, multi-target drugs show lower affinity than one-target-drugs [2, 3]. One of the most promising approaches is the use of synergistic drug combinations therapy able to act on both direct and indirect targets [2, 3]. The use of drug combinations could overcome drug resistance issues associated with high doses of single-hit drugs, and their best efficacy could lead to use a lower drug concentrations reducing unwanted side-effect toxicity [2, 3].

Understanding the effect of individual or combined drugs is critical in clinical studies. An important issue is the low reliability of the cell lines to predict the efficacy of drugs since cell lines did not show to be good models [3]. Thus, computational methods are demanded to deepen the potential role of a drug in a context of pathway cross-talk.

A recent work by Jaeger et al. [6] has proposed a computational method to simulate pathway cross-talk inhibition (PCI) given by individual or combined drugs in BC. In that study the authors considered the pathway cross-talk between two different pathways for shared protein interactions. The authors developed their computational algorithm (PCI index) considering all those KEGG pathways that contain any of the primary targets of the Food and Drug Administration (FDA) approved drugs in BC. No selections on pathways have been performed.

In our recent studies on BC [7, 8], we generated a computational approach to select a network of pathways specific for distinct BC subtypes and quantified their cross-talk. In these works, we focused on a network of pathways composed of interactions among ten pathways; our aim was to study the role of miRNAs regulating pathway interactions in distinct BC subtypes.

In the present study we propose a computational method based on the work of Jaeger et al. [6] and optimized by taking advantage from results of our previous studies [7, 8]. More precisely, here we describe a procedure to build a network of pathways de-regulated for different BC subtypes using gene expression data from The Cancer Genome Atlas (TCGA) and a list of pathways obtained by Ingenuity Pathway Analysis (IPA). We quantified pathway cross-talk with a dissimilarity measure and we assessed potential drug target pathways through PCI. We then applied PCI to quantify the effects of individual or combined drugs in distinct BC subtypes on our network of pathways. Finally, we speculated about the mode of action of FDA approved drugs and new potential drug targets that could decrease the activity of pathway-cross talk and therefore enhance clinical efficacy.

## Methods

### Datasets

We applied the computational approach on four BC subtypes with different diagnostic classification and prognosis [9]: “luminal A” tumors expressing hormone receptors, with a favorable prognosis; “luminal B” tumors expressing hormone receptors and high expression of proliferation genes with a good prognosis although with an increased risk of recurrence; “basal-like” tumors lacking the expression of hormone receptors and HER2 but increased levels of cytokeratin (myoepithelial) (CK 5/6 and CK 17), with shorter observed survival; and “HER2” tumors overexpressing HER2, with the worse survival.

We considered gene expression data from tissue samples studied by IlluminaHiSeq RNASeqV2 and derived from TCGA dataset: 233 BC luminal A samples, 103 BC luminal B samples, 43 BC HER2-overexpressing samples, 74 BC basal samples and 113 normal samples (NS).

We tested the approach with respect to its performance in classifying different subtypes by using an independent testing dataset from Gene Expression Omnibus (GEO) database (GSE58212): 121 luminal A, 69 luminal B, 36 basal and 32 HER2-overexpressing samples.

We validated the approach with respect to its performance in identifying drug target pathways, by using the Matador database [10] that provides interactions between chemicals and proteins. The association between drugs and BC was obtained using 13 drugs already known and approved by FDA for BC [11]: Tamoxifen, Raloxifene, Torimefene, Anastrozole, Letrozole, Exemestane, Capecitabine, Fluorouracil, Gemcitabine, Docetaxel, Vinblastine, Everolimus, and Methotrexate.

## The computational approach

The computational approach consists of seven steps (1. Differential expression analysis; 2. Pathway enrichment; 3. Pathway cross-talk; 4. Classification; 5. Pathway network; 6. Pathway cross-talk inhibition; 7. Drug target pathway network).

All the steps were applied to each of the four BC subtypes. The first four steps were applied 50 times in order to obtain a solid network of de-regulated cross-talking pathways for each BC subtype. More specifically, in order to perform a bootstrapping, we implemented a Monte Carlo cross-validation by randomly selecting some portions of the dataset (60%) to build the training dataset of the classifier and the rest of data (40%) as testing dataset. Step 1, 2, and 3 were applied on the training data set. Step 4 was applied on both training and testing dataset. To avoid problems of unbalanced classes, we randomly generated the same number of samples for each class of BC subtypes and NS.

Then, for each BC subtype, we generated a network-based model of BC subtype pathways (5. Pathway network), and we simulated in silico the drug-induced inhibition of pathways (6. Pathway cross-talk inhibition) focusing on the change of network efficiency.

At the end of the sixth steps, we found potential drug target pathways for each BC subtype, which, if inhibited, can change significantly the network efficiency. These drug targets could be considered for future applications in drug discovery (7. Drug target pathway network).

**Fig. 1:**
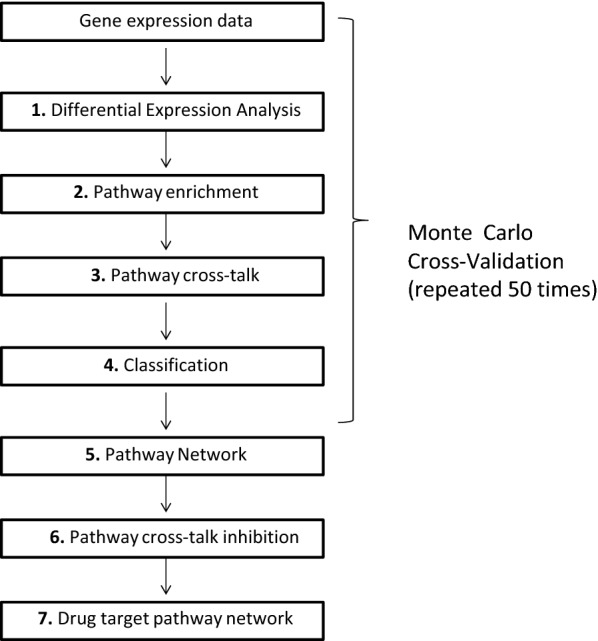
Proposed approach for each subtype

Figure 1 summarizes the proposed computational approach.

We validated our network-based models of BC subtype pathways and BC drug target pathways.

For the first validation task, we assessed the accuracy of using our subtype pathways in classifying four different BC subtypes using the GEO dataset, as data set independent from the TCGA dataset used for the identification of the subtype-derived pathways.

For the second validation task, we evaluated the mechanism of action of 13 FDA approved drugs for BC on our BC subtype drug target pathway network (DTPN). We compared the decrease of pathway cross-talk within the DTCN followed by inhibition of drug target pathways.

### Step 1: differential expression analysis

For each BC subtype, differential expression analysis (DEA) was applied with respect to NS using TCGAbiolinks [12]. In particular, we used the edgeR package from Bioconductor [13] to find differential expressed genes (DEGs) between each BC subtype and NS. For each DEG we calculated the log fold-change between the two conditions and corrected p-values using Benjamini–Hochberg procedure for multiple testing correction [14]. We defined DEGs if the absolute value of log fold change was > 1 and p value < 0.01. This step was applied 50 times on the 50 training datasets producing every time a different list of DEGs.

### Step 2: pathway enrichment analysis

For each BC subtype, we identified a group of pathways significantly enriched with the list of subtype-derived DEGs. The original list of pathways (589) was obtained from IPA. Pathway Enrichment Analysis (PEA) was performed with a Fisher’s test between DEGs and genes within IPA pathways [15]. We defined pathways enriched with DEGs if p-value of Fisher’s test was < 0.01. p-values were corrected using Benjamini–Hochberg procedure for multiple-testing correction [14]. As for the step 1, the step 2 was applied 50 times on the same training dataset of the step 1.

### Step 3: pathway cross-talk

Pathway cross-talk between pathways enriched with subtype-derived DEGs was quantified using a discriminating score (DS) [7, 8]. This score was defined by comparing the mean of the gene expression levels of each pair of pathways enriched with subtype-derived DEGs:

$${\text{DS}} = \frac{{M_{x} - M_{y} }}{{S_{x} + S_{y} }}$$

where $M_{x}$ and $S_{x}$ represent the mean and the standard deviation of the gene expression levels in the pathway *x*, and $M_{y}$ and $S_{y}$ represent the mean and the standard deviation of the gene expression levels in the pathway *y*.

From this step we created a matrix for each sample (BC subtypes and NS) containing a DS value for each pair of pathways enriched with subtype DEGs.

### Step 4: classification

In order to select the best discriminating pairs of cross-talking pathways, we implemented a Random Forest Classification using the R-package [16, 17] to classify each BC subtype versus NS using the DS matrix obtained from step 3 as input of the classifier.

For each combination of pathways we estimated the Area Under Curve (AUC) values by cross-validation method (k-fold cross-validation, k = 10). We used the following parameters: mtry (number of variables randomly sampled as candidates at each split) = sqrt(p), p being the number of variables in the matrix of data; ntree (number of trees grown) = 500.

The classification was performed on the training dataset 50 times for each matrix obtained from the previous three steps. Every time we obtained the top-10 pairs of subtype cross-talking pathways with the best AUC value. Then we validated these pathways on the testing dataset.

In conclusion, for each BC subtype and for all 50 bootstraps we obtained 10 × 50 couples of pathways with the best AUC values validated on the testing dataset.

### Step 5: pathway network

For each BC subtype, by univocally selecting the pairs of cross-talking pathways better discriminating each subtype, we then generated a network-based model of BC subtype pathways, where the nodes of each subtype network represent pathways and the links that connect nodes the pathway cross-talks.

### Step 6: pathways cross-talk inhibition

In a network-based model, the network efficiency is defined as the sum of the reciprocals of the shortest direct path lengths between all pairs of network elements.

If N is the number of network elements and d_i,j_ is the shortest direct path lengths of two elements i and j,

1 $${\text{NE}} = \frac{{\mathop \sum \nolimits_{i \ne j} \frac{1}{d(i,j)}}}{N(N - 1)} \quad {\text{i}},{\text{j}} \in {\text{N}}$$

NE can range from 0 to 1, where 1 means that all nodes interact directly with each other expressing the best efficiency of the network [2].

For each BC subtype, the network efficiency (NE) of the disease was calculated starting from the subtype pathway network, the number of the pathways (nodes) and the shortest direct path lengths between two pathways (e.g. *i*, *j* in *N*).

The efficient drug-induced inhibition of a single pathway can be modelled by the elimination of all direct interactions at the pathway. The corresponding drug effect on the network can be measured by NE as an index of network integrity reduction measuring the drug efficiency [2].

For each BC subtype, we simulated, in silico, the drug-induced inhibition of pathways cross-talk by eliminating, one-by-one, all direct cross-talks. We thus quantified the new NE value of the network, that we called nNE. nNE is thus a function of k, being k the pathway or the combination of the pairs of pathways inhibited in the network.

As effect of this operation, nNE was < NE or > NE, resulting in increasing or decreasing the drug efficiency, respectively.

### Step 7: drug target pathway network

For each BC subtype, we selected those cross-talking pathways from the network, that, if inhibited, caused nNE < NE, thus building a potential DTPN.

Furthermore, from the equation:

2 $${\text{PCI}} = 100 \times \left( {1 - \frac{nNE}{NE}} \right)$$

we quantified the percentage activity of the subtype pathway network that is inhibited, defined as PCI [6].

**Fig. 2:**
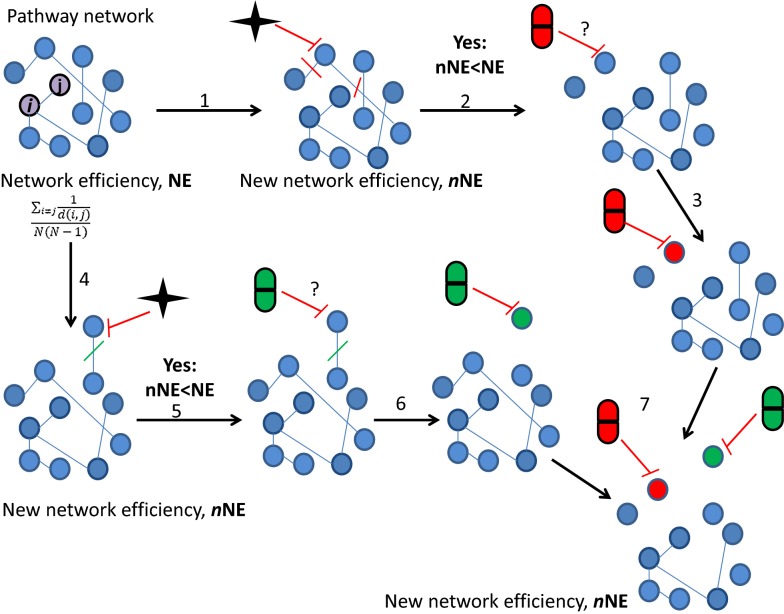
Drug target pathway network. In de-regulated pathway network, the activity of pathway interactions (network efficiency, (NE)) is calculated. *1* and 4 Inhibition of an individual pathway and its interactions. A new-NE (nNE) is calculated. *2* and *5* If nNE < NE the inhibited pathway could be a potential drug target. *3* and *6* integration of drug-pathway associations. *7* nNE is calculated inhibiting two pathways

Figure 2 explains steps 6 and 7 of the proposed approach.

## Validation of DTPN

From the independent GEO dataset GSE58212 (121 luminal A BC, 69 luminal B BC, 36 basal BC and 32 HER2-overexpressing) we considered the gene expression levels belonging to our BC subtype pathway networks. We tested their ability to classify each subtype. We then investigated the role of the top-10 genes that obtained the best classification performance in the perspective of DTPN.

We validated DTPN by assessing if FDA-approved BC drugs have the target pathways within DTPN, and evaluating how these drugs could reduce effectively the NE of the DTPN.

For each BC subtype, we evaluated the association of 13 FDA-approved BC drugs (from Matador database) and DTPN. For this purpose, we applied PEA with a Fisher’s test between gene targets of the drug and genes within IPA pathways. We defined pathways enriched with drug target genes if p-value of Fisher’s test was < 0.01.

For each BC subtype, we measured the effects of the considered drugs, when administered individually and in combination on the DTPN. We quantified this effect by PCI.

## Results

### Luminal A

**Fig. 3:**
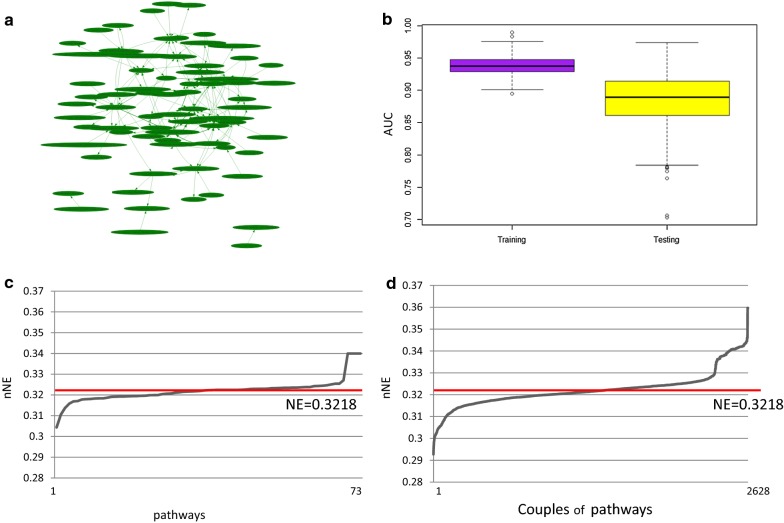
Luminal A BC. **a** Pathway network: nodes represent pathways (73) and edges represent interactions between pathways (157); **b** Boxplot of AUC values for training and testing dataset; **c** Trend of new network efficiency (nNE) calculated after removal of each of the 73 individual pathways; **d** nNE values calculated after removal of all combinations of 2628 couples of pathways. Red lines represent the efficiency of the original network (NE)

For the Luminal A we found a pathway network composed of 73 individual pathways and 157 pathway cross-talks (Fig. 3a). AUC for this pathway network was 0.93 in the TCGA training dataset and 0.88 in the TCGA testing dataset, respectively (Fig. 3b).

The efficiency of the pathway network (NE) was 0.3218. nNE, calculated after the drug-induced in silico inhibition of each single pathway (among the 73) or each combination of pair of pathways (among the 2628) is shown in Fig. 3c and d, respectively. The inhibition of 34/73 individual pathways reduces NE (red line) of the pathway network with nNE values that ranges from 0.3043 to 0.3217 (Fig. 3c). The inhibition of 1388 combinations (among 2628) reduces the NE (red line) of the pathway network with values that range from 0.2927 to 0.3217 (Fig. 3d). Thus, the inhibition of couples of pathways of the cloud seems to reduce the efficiency more than individual pathway.

Additional file 1 shows nNE values in the luminal A network after inhibition of individual and pairs pathway(s). The top-10 pathways, which, if inhibited, have resulted in a better efficiency reduction of the network are: ‘LXR/RXR Activation’, ‘Extrinsic Prothrombin Activation Pathway’, ‘Estrogen Receptor Signaling’, ‘Human Embryonic Stem Cell Pluripotency’, ‘Ethanol Degradation IV’, ‘RAR Activation’, ‘Fatty Acid oxidation’, ‘Bladder Cancer Signaling’, ‘Factors Promoting Cardiogenesis in Vertebrates’ and ‘Glioma Invasiveness Signaling’. ‘LXR/RXR Activation’ plays the most important role since its single inhibition reduces the network efficiency of 5.5% (PCI). The inhibition of both ‘LXR/RXR Activation Pathway’ and ‘Extrinsic and Prothrombin Activation Pathway’ is the most effective reducing the efficiency of the network of 9% (PCI). In line with the use of synergistic drug combinations, drugs acting on both ‘LXR/RXR Activation Pathway’ and ‘Extrinsic and Prothrombin Activation Pathway’ could be more effective.

DTPN of Luminal A was built considering only the 34 pathways of the network that if inhibited, reduced the NE (nNE < NE).

Starting from the 13 FDA approved drugs for BC (fluorouracil, anastrozole, capecitabine, docetaxel, exemestane, fulvestrant, gemcitabine, letrozole, methotrexate, raloxifene, tamoxifene, toremifene, and vinblastine) we obtained 8 drugs that interact with the DTPN of Luminal A (capecitabine, fulvestrant, gemcitabine, methotrexate, raloxifene, tamoxifen, toremifene and vinblastine) (Table 1).

**Table 1:** Drug-pathway association in the DTPN of Luminal A subtype

Drug	Pathway	nNE and PCI (vs NE = 0.3218)
Capecitabine	Eicosanoid	0.3194 (PCI 0.75%)
Fulvestrant	Bladder cancer signaling (1); estrogen receptor (2); regulation of cellular mechanics by calpain protease (3)	(1) 0.3179 (PCI 1.18%) (2) 0.3137 (PCI 2.50%) (3) 0.3198 (PCI 0.59%) (1–2) 0.3094 (PCI 3.84%) (1–3) 0.3159 (PCI 1.81%) (2–3) 0.3115 (PCI 3.18%) (1–2–3) 0.3071 (PCI 4.56%)
Gemcitabine	Regulation of cellular mechanics by calpain protease	0.3198 (PCI 0.59%)
Methotrexate	FXR/RXR activation	0.3193 (PCI 0.75%)
Raloxifene	Estrogen receptor (1); RAR activation (2)	(1) 0.3137 (PCI 2.50%) (2) 0.3170 (PCI 1.47%) (1–2) 0.3098 (PCI 3.70%)
Tamoxifen	Estrogen receptor (1); factors promoting cardiogenesis in vertebrates (2); human embryonic stem cell pluripotency (3); RAR activation (4)	(1) 0.3137 (PCI 2.50%) (2) 0.3180 (PCI 1.17%) (3) 0.3157 (PCI 1.86%) (4) 0.3170 (PCI 1.47%) (1–2) 0.3092 (PCI 3.88%) (1–3) 0.3071 (PCI 4.56%) (1–4) 0.3098 (PCI 3.70%) (2–3) 0.3098 (PCI 3.70%) (2–4) 0.3129 (PCI 2.74%) (3–4) 0.3103 (PCI 3.56%) (1–2–3) 0.3003 (PCI 6.68%) (2–3–4) 0.3025 (PCI 5.98%) (3–4–1) 0.3051 (PCI 5.18%) (1–2–3–4) 0.2941 (PCI 8.59%)
Toremifene	FXR/RXR activation (1); pregnenolone biosynthesis (2)	(1) 0.3193 (PCI 0.75%) (2) 0.3192 (PCI 0.79%) (1–2) 0.3165 (PCI 1.61%)
Vinblastine	Axonal guidance signaling (1); FXR/RXR activation (2)	(1) 0.3216 (PCI 0.05%) (2) 0.3193 (PCI 0.75%) (1–2) 0.3190 (PCI 0.86%)

In particular, the inhibition of the ‘Estrogen Receptor Signaling’ reduces the NE of 2.50%. This inhibition could be obtained using three FDA approved drug (fulvestrant, raloxifene and tamoxifen) that have a significant number of target genes belonging to this pathway.

The ‘FXR/RXR Activation Pathway’ if inhibited, reduces the NE of 0.75%, and it is target of three drugs (methotrexate, toremifene and vinblastine). The ‘RAR Activation Pathway’, if inhibited, reduces the NE of 1.5% and it is target of two drugs (raloxifene and tamoxifen). The ‘Regulation of Cellular Mechanics by Calpain Protease’ pathway, if inhibited, reduces the NE of 0.6% and is target of two drugs (fulvestrant and gemcitabine).

Among the drug target pathways found in our DTPN, we found 5/34 pathways that were ranked as part of the top 10 pathways, playing the major role in the reduction of NE. These pathways are ‘Estrogen Receptor’ targeted by tamoxifen and raloxifene; ‘Human Embryonic Stem Cell Pluripotency’ targeted by tamoxifen; ‘RAR Activation Pathway’ targeted by raloxifene and tamoxifen; ‘Bladder Cancer Signaling’ targeted by fulvestrant; ‘Factors Promoting Cardiogenesis in Vertebrates’ targeted by tamoxifen. In particular, tamoxifen acting on 4 pathways reduces the NE of 8.59%, thus it is very effective.

FDA-approved drugs do not seem to act on ‘LXR/RXR Activation’, ‘Extrinsic Prothrombin Activation’; ‘Ethanol Degradation IV’, ‘Fatty Acid oxidation’ and ‘Glioma Invasiveness Signaling’. In particular the first two pathways, according to our findings, could be potential dug targets for anticancer drug treatment.

Furthermore, considering FDA-approved drugs, our study confirms that tamoxifen is the drug with the best efficacy on the pathway network since it is able alone to inhibit four pathways (‘Estrogen receptor’, ‘Human Embryonic Stem Cell Pluripotency’, ‘Factors Promoting Cardiogenesis in Vertebrates’ and ‘RAR activation’).

### Luminal B

**Fig. 4:**
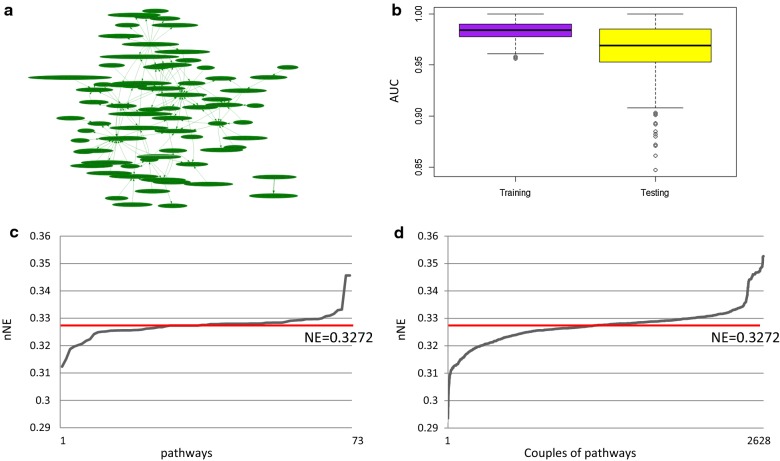
Luminal B. **a** Pathway network: nodes represent pathways (73) and edges represent interactions between pathways (129); **b** Boxplot of AUC values for training and testing dataset; **c** Trend of new network efficiency (nNE) calculated after removal of individual pathways; **d** Trend of nNE values calculated after removal of all combinations of couples of pathways. Red lines represent the efficiency of the original network (NE)

In luminal B we found a pathway network composed of 73 individual pathways and 129 pathway cross-talks (Fig. 4a). In the TCGA training dataset mean AUC was 0.98 and 0.96 in the TCGA testing dataset (Fig. 4b).

The efficiency of the pathway network was 0.3272. Figure 4c and d show the trend of nNE in case of inhibition of 73 individual pathways or each combination of pairs of pathways (among 2628), respectively.

The inhibition of 27/73 individual pathways reduces the NE of the pathway network with values that range from 0.3123 to 0.3270 (Fig. 4c). The inhibition of 1199/2628 combinations of pairs of pathways reduces the NE with values that range from 0.2935 to 0.32720 (Fig. 4d). As for luminal A, also in luminal B the inhibition of pairs of pathways of the pathway network reduce the efficiency more than individual pathway.

Additional file 2 shows nNE values in the luminal B after the inhibition of individual and combined pathway(s). The top-10 pathways, which if inhibited, lead to the best reduction of network efficiency include: ‘Cell Cycle Control of Chromosomal Replication’, ‘P2Y Purigenic Receptor Signaling Pathway’, ‘Growth Hormone Signaling’, ‘Epithelial Adherens Junction Signaling’, ‘Regulation of the Epithelial-Mesenchymal Transition Pathway’, ‘Mitotic Roles of Polo-Like Kinase’, ‘Tight Junction Signaling’, ‘Role of BRCA1 in DNA Damage Response’, ‘Cellular Effects of Sildenafil (Viagra)’ and ‘Cell Cycle: G2/M DNA Damage Checkpoint Regulation’. The inhibition of ‘Cell Cycle Control of Chromosomal Replication’ plays an important role reducing the efficiency of 4.5% (PCI). The inhibition of both ‘Growth Hormone Signaling’ and ‘Regulation of the Epithelial-Mesenchymal Transition Pathway’ plays the most important role reducing the efficiency of the network of 10% (PCI).

DTPN of luminal B was built considering the 27 pathways that inhibited reduced the NE. We obtained 4 BC drugs (among the 13 FDA approved) (docetaxel, fulvestrant, raloxifene, and tamoxifen) that interact with the DTPN of luminal B (Table 2).

**Table 2:** Drug-pathway association in the DTPN of luminal B subtype

Drug	Pathway	nNE and PCI (vs NE = 0.3272)
Docetaxel	Germ cell-sertoli cell junction signaling (1); epithelial adherens junction signaling (2)	(1) 0.3256 (PCI 0.46%) (2) 0.3196 (PCI 2.30%) (1–2) 0.3180 (PCI 2.80%)
Fulvestrant	Estrogen-mediated S-phase entry	0.327093 (PCI 0.03%)
Raloxifene	Estrogen-mediated S-phase entry	0.327093 (PCI 0.03%)
Tamoxifen	Wnt/catenin signaling (1); regulation of the epithelial-mesenchymal transition pathway (2); estrogen-mediated S-phase entry (3)	(1) 0.3254 (PCI 0.53%) (2) 0.3201 (PCI 2.14%) (3) 0.3270 (PCI 0.03%) (1–2) 0.3182 (PCI 2.73%) (1–3) 0.3251 (PCI 0.64%) (3–2) 0.3196 (PCI 2.29%) (1–2–3) 0.3174 (PCI 3.06%)

In particular, the ‘Estrogen-mediated S-phase Entry Pathway’ is target of three approved drugs (fulvestrant, raloxifene and tamoxifen). The ‘Epithelial Adherens Junction Signaling Pathway’, if inhibited, reduces the NE of 2.3% and it is target of docetaxel. The ‘Germ Cell-Sertoli Cell Junction Signaling Pathway’, if inhibited, reduces the NE of 0.4% and it is target of docetaxel. The ‘Regulation of the Epithelial-Mesenchymal Transition Pathway’, if inhibited, reduces the NE of 2.1% and it is target of tamoxifen. The ‘Wnt/catenin Signaling Pathway’, if inhibited, reduces the NE of 0.5% and it is target of tamoxifen.

Among the drug target pathways found in luminal B DTPN, we found 2/27 pathways that were ranked as to be part of the top 5 pathways playing the major role in the reduction of NE. These pathways are ‘Epithelial Adherens Junction Signaling’ and ‘Regulation of the Epithelial-Mesenchymal Transition Pathway’ targeted by docetaxel and tamoxifen, respectively.

FDA-approved drugs do not seem to act on ‘Cell Cycle Control of Chromosomal Replication’, ‘P2Y Purigenic Receptor Signaling Pathway’ and ‘Growth Hormone Signaling’; pathways that according our findings were the best potential drug targets. In particular, tamoxifen acting on three pathways reduces the NE of 3%.

Furthermore, considering FDA-approved drugs our study demonstrates that docetaxel is the drug with the best action on pathway network since is able to inhibit ‘Epithelial Adherens Junction Signaling’.

### HER2-overexpressing BC

**Fig. 5:**
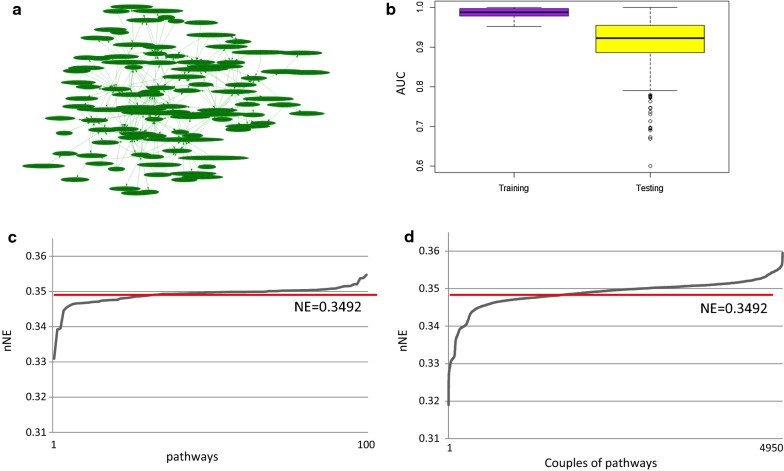
HER2-overexpressing BC (HER2). **a** Pathway network: nodes represent pathways (100) and edges represent interactions between pathways (222); **b** Boxplot of AUC values for training and testing dataset; **c** Trend of new network efficiency (nNE) calculated after removal of individual pathways; **d** nNE values calculated after removal of all combinations of couples of pathways. Red lines represent the efficiency of the original network (NE)

We found a pathway network in HER2 BC composed of 100 individual pathways and 222 pathway cross-talks (Fig. 5a). Mean AUC was 0.98 and 0.91 in the TCGA training and testing dataset, respectively (Fig. 5b).

The efficiency of the pathway network in HER2 was 0.3492. Figure 5c and d show nNE in case of inhibition of individual pathways or each combination of pairs of pathways, respectively. The inhibition of 39/100 individual pathways reduces the NE of the pathway network with values that range from 0.3309 to 0.3492 (Fig. 5c). The inhibition of 2271/4950 combinations of pathways reduces the NE of the pathway network with values that ranges from 0.3189 to 0.3492 (Fig. 5d). It is confirmed that the inhibition of couples of pathways of the pathway network reduces the efficiency more than individual pathway.

Additional file 3 shows nNE values after inhibition of individual and combined pathway(s) in HER2. The top-10 pathways, which if inhibited, have resulted in a better efficiency reduction of the network include: ‘Role of BRCA1 in DNA Damage Response’, ‘NAD biosynthesis II (from tryptophan)’, ‘Protein Kinase A Signaling’, ‘Ephrin Receptor Signaling’, ‘Growth Hormone Signaling’, ‘Cellular Effects of Sildenafil (Viagra)’, ‘Human Embryonic Stem Cell Pluripotency’, ‘Axonal Guidance Signaling’, ‘CXCR4 Signaling’ and ‘Acute Phase Response Signaling’. The inhibition of ‘Role of BRCA1 in ‘‘DNA Damage Response’ an important role reducing the efficiency of 5.2% (PCI). The inhibition of both ‘NAD biosynthesis II (from tryptophan)’ and ‘Role of BRCA1 in DNA Damage Response’ plays a more important role reducing the efficiency of the network of 8% (PCI).

DTPN was built considering 39 pathways whose inhibition reduces the efficiency of the network. Starting from the 13 BC drugs, we obtained five drugs (fluoracil, docetaxel, fulvestrant, methotrexate, and tamoxifen) that interact with the HER 2 DTPN (Table 3).

**Table 3:** Drug-pathway association in the DTPN of HER2 subtype

Drug	Pathway	nNE and PCI (vs NE = 0.3492)
Fluoracil	Salvage pathways of pyrimidine deoxyribonucleotides	0.3482 (PCI 0.27%)
Docetaxel	Epithelial adherens junction signaling	0.3485 (PCI 0.17%)
Fulvestrant	Bladder cancer signaling	0.3473 (PCI 0.51%)
Methotrexate	Salvage pathways of pyrimidine deoxyribonucleotides	0.3482 (PCI 0.27%)
Tamoxifen	Human embryonic stem cell pluripotency (1); regulation of the epithelial-mesenchymal transition (2)	(1) 0.3464 (PCI 0.78%) (2) 0.3490 (PCI 0.02%) (1–2) 0.3462 (PCI 0.84%)

In particular, the ‘Salvage Pathways of Pyrimidine Deoxyribonucleotides’ is target of two FDA-approved BC drugs (fluoracil, and methotrexate). The ‘Bladder Cancer Signaling Pathway’, if inhibited, reduces the NE of 0.5% and it is target of fulvestrant. The ‘Epithelial Adherens Junction Signaling Pathway’, if inhibited, reduces the NE of 0.2%, and it is target of docetaxel. The ‘Human Embryonic Stem Cell Pluripotency pathway’, if inhibited, reduces the NE of 0.8% and it is target of tamoxifen. The ‘Regulation of the Epithelial-Mesenchymal Transition Pathway’ if inhibited, reduces the NE of 0.1% and it is target of tamoxifen. The ‘Regulation of the Salvage Pathways of Pyrimidine Deoxyribonucleotides’, if inhibited, reduces the NE of 0.2% and it is target of fluoracil, and methotrexate.

Among the drug target pathways found in our DTPN we found 1 pathway that was ranked as to be part of the top 10 pathways playing the major role in the reduction of NE (‘Human Embryonic Stem Cell Pluripotency’).

FDA-approved drugs do not seem to act on ‘Role of BRCA1 in DNA Damage Response Pathway’ and ‘NAD biosynthesis II (from tryptophan)’, pathways that, according to our findings, were the best potential drug targets.

Furthermore, considering FDA-approved drugs, our study confirms that tamoxifen is the drug with the best action on pathway network since it is able to inhibit ‘Human Embryonic Stem Cell Pluripotency’.

### Basal BC

**Fig. 6:**
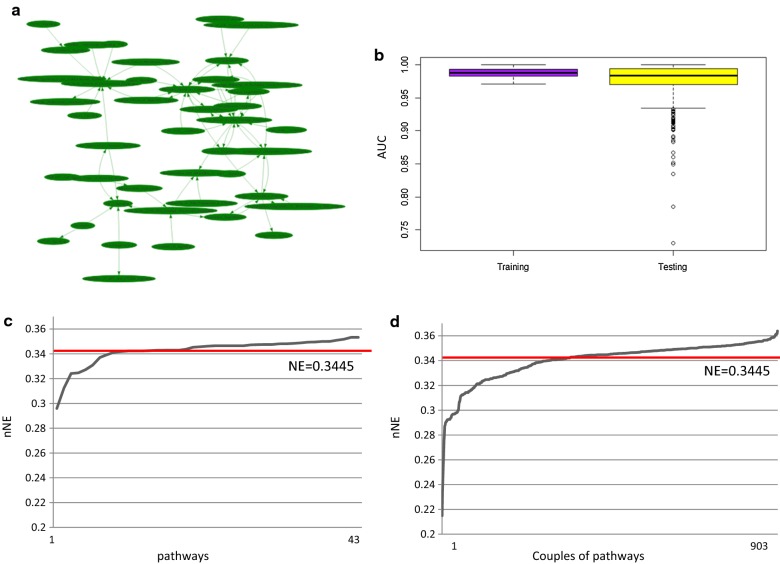
Basal. **a** Pathway network: nodes represent pathways (43) and edges represent interactions between pathways (74); **b** Boxplot of AUC values for training and testing dataset; **c** Trend of new network efficiency (nNE) calculated after removal of individual pathways; **d** Trend of nNE values calculated after removal of all combinations of couples of pathways. Red lines represent the efficiency of the original network (NE)

We found a pathway network in basal BC composed of 43 individual pathways and 74 pathway cross-talks (Fig. 6a). In the TCGA training dataset the mean AUC was 0.98 and 0.97 in the TCGA testing dataset (Fig. 6b).

The efficiency of the pathway network in basal BC was 0.3445. nNE in case of the single inhibition of the 43 individual pathways or each combination of pairs of pathways are shown in Fig. 6c and d, respectively. The inhibition of 19/43 of individual pathways reduces the NE of the pathway network with values that ranges from 0.2959 to 0.3443 (Fig. 6c). The inhibition of 416/903 combinations of pathways reduces the NE of the pathway network with values that ranges from 0.2148 to 0.3445 (Fig. 6d). The inhibition of couples of pathways is confirmed to reduce the efficiency more than individual pathway.

Additional file 4 shows nNE values after inhibition of individual and combined pathway(s) in basal BC. The top-10 pathways which, if inhibited, have resulted in a better efficiency reduction of the network include: ‘EIF2 Signaling’, ‘Mismatch Repair in Eukaryotes’, ‘Aryl Hydrocarbon Receptor Signaling’, ‘Cell Cycle Control of Chromosomal Replication’, ‘Cell Cycle: G2/M DNA Damage Checkpoint Regulation’, ‘Role of BRCA1 in DNA Damage Response’, ‘Noradrenaline and Adrenaline Degradation’, ‘Histamine Degradation’, ‘Tryptophan Degradation X (Mammalian, via Tryptamine)’ and ‘Dopamine Degradation’. The inhibition of ‘EIF2 Signaling’ plays a more important role reducing the efficiency of 14% (PCI). The inhibition of both ‘Mismatch Repair in Eukaryotes’ and ‘Cell Cycle:G2/M DNA Damage Checkpoint Regulation’ plays a more important role reducing the efficiency of the network of 37% (PCI).

DTPN of basal BC was built considering 19 pathways that reduced NE. We obtained 6 drugs (fluoracil, capecitabine, fulvestrant, methotrexate, raloxifene and tamoxifen) that interact with the DTPN in basal BC (Table 4).

**Table 4:** Drug-pathway association in the DTPN of basal BC subtype

Drug	Pathway	NE	nNE and PCI (vs NE = 0.3445)
Fluorouracil	Aryl hydrocarbon receptor signaling (1); salvage pathways of pyrimidine deoxyribonucleotides (2)	0.3445	(1) 0.3241 (PCI 5.9%) (2) 0.3425 (PCI 0.56%) (1–2) 0.3208 (PCI 6.86%)
Capecitabine	Triacylglycerol degradation	0.3445	0.3429 (PCI 0.46%)
Fulvestrant	Aryl hydrocarbon receptor signaling	0.3445	0.3241 (PCI 5.9%)
Methotrexate	Salvage pathways of pyrimidine deoxyribonucleotides	0.3445	0.3425 (PCI 0.56%)
Raloxifene	Aryl hydrocarbon receptor signaling	0.3445	0.3241 (PCI 5.9%)
Tamoxifene	Aryl hydrocarbon receptor signaling	0.3445	0.3241 (PCI 5.9%)

In particular, the ‘Salvage Pathways of Pyrimidine Deoxyribonucleotides’ is target of two FDA-approved drugs (fluorouracil, and methotrexate) and ‘Aryl Hydrocarbon Receptor Signaling’ of 4 FDA-approved drugs (fluorouracil, fulvestrant, raloxifene, and tamoxifene). The ‘Aryl Hydrocarbon Receptor Signaling’, if inhibited, reduces the NE of 5%. The ‘Salvage Pathways of Pyrimidine Deoxyribonucleotides’, if inhibited, reduces the NE of 0.5%. The ‘Triacylglycerol Degradation Pathway’, if inhibited, reduces the NE of 0.4% and it is target of capecitabine.

Among drug target pathways found in our DTPN we found 1 pathway of the top 5 pathways playing the major role in the reduction of NE (‘Aryl Hydrocarbon Receptor Signaling’). In particular, fluorouracil acting on 2 pathways reduces the NE of 6.86%, thus it is very effective.

The considered FDA approved drugs for BC do not seem to act on ‘EIF2 Signaling’ and ‘Mismatch Repair in Eukaryotes’, pathways that, according to our findings, could be interesting potential drug targets.

### Validation of DTPN

To classify luminal A, we considered the expression levels of 2693 genes from the independent GEO dataset GSE58212 and belonging to our luminal A pathway network. We found PRC1 (AUC = 0.862), CCNB2 (AUC = 0.847), BIRC5 (AUC = 0.838), PTTG1 (AUC = 0.831), CCNA2 (AUC = 0.827), E2F1 (AUC = 0.826), KIF11 (AUC = 0.826), CDC25A (AUC = 0.823), UBE2C (AUC = 0.82), and CDK1 (AUC = 0.817) as the top 10 genes obtaining the best classification performance. Among them, 7/10 genes play a crucial role in luminal A DTPC. PRC1, CCNB2, PTTG1, KIF11, and CDC25A belong to ‘Mitotic Roles of Polo-Like Kinase’ pathway. The inhibition of this pathway can reduce the NE of luminal A of 1% (PCI). CCNA2 and CDK1 belong to ‘Regulation of Cellular Mechanics by Calpain Protease’ pathway. Its inhibition can reduce the NE of luminal A of 0.6% (PCI).

In luminal B BC we considered the expression levels of 2158 genes from the GEO dataset belonging to our luminal B pathway network. We found CX3CL1 (AUC = 0.779), KIF11 (AUC = 0.777), PTTG1 (AUC = 0.766), PRC1 (AUC = 0.761), CCNA2 (AUC = 0.757), ACTG2 (AUC = 0.756), CCNB1 (AUC = 0.755), RND3 (AUC = 0.752), KIF23 (AUC = 0.751), and CLDN8 (AUC = 0.749) as the top 10 genes. Among them, 6/10 genes play a crucial role in luminal B DTPN. KIF11, CCNB1, PTTG1, PRC1, and KIF23 belong to ‘Mitotic Roles of Polo-Like Kinase’ pathway, whose inhibition can reduce the NE of luminal B of 2% (PCI). ACTG2 belongs to ‘Tight Junction Signaling’ pathway. Its inhibition can reduce the NE of 1.7% (PCI).

In HER2 BC we considered the expression levels of 1172 genes from the GEO dataset belonging to 100 pathways of our HER2 pathway network. We found ESR1 (AUC = 0.811), E2F1 (AUC = 0.78), RARG (AUC = 0.769), PNMT (AUC = 0.758), PLCB3 (AUC = 0.757), ERBB2 (AUC = 0.741), MYL5 (AUC = 0.729), PPP1R10 (AUC = 0.729), BCL2 (AUC = 0.727), and WNT3 (AUC = 0.724), as the top 10 genes.

Among them 6/10 genes play a crucial role in DTPN in HER2. E2F1 belongs to ‘Role of BRCA1 in DNA Damage Response’ and ‘Growth Hormone Signaling’ pathways. These pathways are involved in HER2 DTPN since their inhibition can reduce the NE of 5.3 and 1.1% (PCI), respectively.

PLCB3, ERBB2, MYL5, and WNT3 belong to ‘Axonal Guidance Signaling’ pathway, whose inhibition can reduce the NE of HER2 of 0.8% (PCI). PPP1R10 belongs to ‘Protein Kinase A Signaling’. Its inhibition can reduce the NE of 2.8% (PCI).

Furthermore, ESR1 is targeted by fulvestrant, toremifene, raloxifene, and tamoxifen; ERBB2 is targeted by tamoxifen, and BCL2 is targeted by fulvestrant, gemcitabine, docetaxel and tamoxifen.

In basal BC we considered expression levels of 1196 genes from the GEO dataset belonging to 43 pathways of basal pathway network. We found RHOB (AUC = 0.946), FBP1 (AUC = 0.942), RARA (AUC = 0.911), PPP1R14C (AUC = 0.909), E2F3 (AUC = 0.905), F7 (AUC = 0.904), RND1 (AUC = 0.903), ESR1 (AUC = 0.902), CDC20 (AUC = 0.892), and CCNE1 (AUC = 0.892), as the top 10 genes.

RARA, ESR1, and CCNE1 belong to ‘Aryl Hydrocarbon Receptor Signaling’ pathway whose inhibition can reduce the NE of 6% (PCI). E2F3 belongs to ‘Role of BRCA1 in DNA Damage Response’ pathway. This pathway is involved in basal DTPN since its inhibition can reduce the NE of 4% (PCI).

### DTPN pathways in BC subytpes

We have constructed a model of BC heterogeneity based on different subtype pathway networks. Table 5 shows de-regulated pathways in common in all subtypes, which can be considered as responsible for the initial stages of BC, and those de-regulated pathways present only in one subtype, specific of the behaviour of that subtype.

**Table 5:** Pathways in common in all breast cancer subtypes and specific for each subtype as derived from the proposed network approach

Common to all	Luminal A	Luminal B	HER2	Basal
Assembly of RNA polymerase II complex	Cell cycle regulation by BTG family proteins	Antioxidant action of vitamin C	Actin cytoskeleton signaling	*Aryl Hydrocarbon Receptor Signaling*
Axonal guidance signaling	Chemokine signaling	Germ cell-sertoli cell junction signaling	Adrenergic signaling	eNOS signaling
Coagulation system	Chondroitin sulfate biosynthesis	HMGB1 signaling	Breast cancer regulation by Stathmin1	Fatty acid oxidation I
Colorectal cancer metastasis signaling	chondroitin sulfate biosynthesis (late stages)	IL-6 signaling	cAMP-mediated signaling	Gluconeogenesis I
EIF2 signaling	Ephrin B signaling	Linolenate biosynthesis II (animals)	Cardiac hypertrophy signaling	Glycolysis I
Ethanol degradation II	Granulocyte adhesion and diapedesis	Antioxidant action of vitamin C	Caveolar-mediated endocytosis signaling	IL-1 signaling
Ethanol Degradation IV	Heparan sulfate biosynthesis	Germ cell-sertoli cell junction signaling	Corticotropin Releasing Hormone Signaling	Mitochondrial Dysfunction
Extrinsic prothrombin activation pathway	Heparan sulfate biosynthesis (late stages)	HMGB1_Signaling	CREB signaling in neurons	mTOR signaling
Fatty acid-oxidation	*LXR/RXR activation*	IL-6 signaling	DNA damage-induced 14–3–3 signaling	Phenylalanine Degradation IV (Mammalian, via Side Chain)
HIF1 signaling	Pancreatic adenocarcinoma signaling	Linolenate biosynthesis II (animals)	Endothelin-1 signaling	Phototransduction pathway
Histamine degradation	Pregnenolone biosynthesis		Gap junction signaling	PI3K/AKT signaling
Noradrenaline and adrenaline degradation	Regulation of cellular mechanics by calpain protease		GDNF family ligand–receptor interactions	
Oxidative ethanol degradation III	Semaphorin signaling in neurons		Glycine betaine degradation	
Putrescine degradation III	Superoxide radicals degradation		Glycogen degradation II	
Tryptophan degradation X (mammalian, via tryptamine)			Induction of apoptosis by HIV1	
			Leptin signaling in obesity	
			Macropinocytosis signaling	
			*NAD biosynthesis II (from tryptophan)*	
			Ovarian cancer signaling	
			Relaxin signaling	
			RhoGDI signaling	
			Role of IL-17A in psoriasis	
			Role of tissue factor in cancer	
			Sperm motility	
			Synaptic long term depression	
			tRNA splicing	
			Tryptophan degradation to 2-amino-3-carboxymuconate semialdehyde	
			TWEAK Signaling	

In italics pathways that are target of FDA-approved drugs for breast cancer are indicated

We can observe in particular that: (i) ‘LXR/RXR Activation Pathway’, that in our analyses emerged as a new potential drug target for luminal A, is specific for this subtype; (ii) ‘NAD biosynthesis II (from tryptophan)’ pathway, that according to our findings was found the best potential drug target in HER2, is specific for HER2 subtype; and (iii) ‘Aryl Hydrocarbon Receptor Signaling’, that is targeted by 4 FDA-approved drugs (fluorouracil, fulvestrant, raloxifene, and tamoxifene) is specific of basal subtype.

## Discussion

The formation of new alternative signaling pathways upon drug treatment is one of the main causes of inefficacy or development of drug resistance in cancer. The study of alternative signalling through pathway cross-talk can help drug-development strategies. Therefore, in this work, we optimized a computational method to investigate the role of pathways target of FDA approved drugs or new drugs that specifically addresses this issue. Our in silico method is based on the identification and quantification of the pathway cross-talk for distinct cancer subtypes. The effects of drugs inhibiting individual or combined pathways can be simulated and measured with the purpose to find new potential drug-targets or synergistic combination drugs.

Most works that study drug-development approaches use network models. Usually the most used methods to examine the effects of drugs involve protein–protein interactions, metabolic control analysis or neural network [18–21]. Nevertheless, most of these methods are focused on the detection of drugs that have only one target (single hit) and often are not able to reveal the pathway interactions in an effective way [2]. Furthermore, these methods show to depend on a large number of parameters [2]. Pathway cross-talk can be an ideal framework to assess pathway interactions that could originate phenomena of resistance or inefficacy of the drug. The investigation of drug-induced modifications on the different levels of pathway cross-talk can improve the knowledge of drug effects and potential drug targets.

Although based by the computational method of Jaeger et al. [6], our application presents several differences: (1) the Jaeger’s work was focused on cross-talk due to the presence of overlapping genes between different pathways, we instead explore cross-talk as regulatory interactions among distinct pathways; (2) in Jaeger’s work all KEGG pathways were considered, we instead made a selection on pathways based on their different activity in different cancer subtype *vs* normal tissues. In line with the last scenario, the computational method of Jaeger et al. is suitable to model static pathway cross-talks, while our method could be used to represent dynamic disease progression.

Using Monte-Carlo cross validation and classification, we built networks of cross-talking pathways able to classify with high performance cancer subtypes versus NS. We inhibited pathway cross-talks in such subtype-derived networks and identified a number of pathways that could be potential targets of drugs by measuring drug ability in reducing the network efficiency.

We applied our network-based model of cross-talking pathways to the BC subtype gene expression level of an independent GEO dataset, to test independently the ability of the pathway networks to classify BC subtypes. We found that the top 10 genes achieving the best classification performance belong to the expected cross-talking pathways.

We then associated some drugs already approved by FDA for BC with the networks of cross-talking pathways. The association was done considering the number of known drug-target proteins mapped on pathways and selecting potential synergistic combination of drugs. In general, the inhibition of pairs of pathways of the pathway network reduces the network efficiency more than the inhibition of individual pathways. The considered FDA approved drugs for BC act on several pathways included in the networks of cross-talking pathways. However, they do not act on other pathways that, according to our findings, could be interesting potential drug targets in BC.

In luminal A, we found that the individual inhibition of ‘LXR/RXR Activation’ pathway reduces the efficiency of the network of 5.6%. LXRs are nuclear receptors (NRs) involved in cholesterol, glucose, fatty acid metabolism and inflammatory responses. NRs are a family of transcription factors that bind to respond to lipophilic signaling molecules (ligand) regulating downstream effectors. They also represent one of the most important drug-development targets, since the designing of synthetic compounds that mimic the functions of ligands can selectively modulate the activity of NRs [22]. Selective estrogen receptor modulators (SERMs) and aromatase inhibitors (AI) are examples of synthetic compounds, which block the production of estrogen [23]. LXRs ligands on prostate cancer reported an effect on cell proliferation and cell cycle, acting on p27 and SKP2 [24]. In BC the effects of LXRs ligands seem to be slightly different, as there is a decrease of SKP2 but not of p27 [25]. However, there are conflicting reports about the potential role of ‘LXR/RXR Activation’ pathway in BC [25–28]. In our diagnostic pathway cloud, synergistic drug combinations acting on: (1) ‘LXR/RXR Activation’ and ‘Extrinsic and Prothrombin Activation’ Pathways; (2) ‘LXR/RXR Activation’ and ‘Estrogen Receptor Signaling’; (3) ‘LXR/RXR Activation pathway’ and ‘RAR Activation’ reduce of almost 9% the efficiency of the network in luminal A. To date, synergistic drug combinations performing on these pathways are not reported. Therefore, in luminal A we suggest LXR/RXR Activation as drug-pathway target combined with a drug acting on ‘Extrinsic and Prothrombin Activation’ Pathway, ‘Estrogen Receptor Signaling’ or ‘RAR Activation’.

In luminal B subtype we found that the individual inhibition of ‘Cell Cycle Control of Chromosomal Replication’ reduces the efficiency of the network of 4.5% while, in our pathway network, synergistic drug combinations acting on: (1) ‘Growth Hormone Signaling’ and ‘Regulation of the Epithelial-Mesenchymal Transition’ Pathways; (2) ‘Mitotic Roles of Polo-Like Kinase’ and ‘Cell Cycle Control of Chromosomal Replication’; (3) ‘Cell Cycle Control of Chromosomal Replication’ and ‘Growth Hormone Signaling’ reduce of almost 10% the efficiency of the network. To date, synergistic drug combinations targeting on these pathways are not presented. Regarding FDA approved drugs, we confirm docetaxel and tamoxifen as promising candidates for synergistic drug combinations since they can modulate ‘Epithelial Adherens Junction Signaling’ and ‘Regulation of the Epithelial-Mesenchymal Transition Pathway’, respectively. Previous studies showed that a combination therapy of these drugs in BC cell lines increases the anti-proliferative effects of single agents [29, 30].

In HER2 subtype we found that the inhibition of BRCA1 in ‘DNA Damage Response’ reduces the efficiency of 5.2% while, in our pathway network, synergistic drug combinations acting on ‘NAD biosynthesis II (from tryptophan)’ and ‘Role of BRCA1 in DNA Damage Response’ reduce the efficiency of 8%.

In basal subtype we found that the inhibition of EIF2 Signaling reduces the efficiency of 8.5%. While, in our diagnostic pathway cloud, synergistic drug combinations acting on ‘Mismatch Repair in Eukaryotes’ and ‘Cell Cycle:G2/M DNA Damage Checkpoint Regulation’ reduce the efficiency of the network of 37%. Regarding FDA-approved drugs, we suggest fulvestrant, or fluorouracil and/or capecitabine as promising candidates for synergistic drug combinations since they can modulate ‘Epithelial Salvage Pathways of Pyrimidine Deoxyribonucleotides’, ‘Aryl Hydrocarbon Receptor Signaling’ and ‘Triacylglycerol Degradation’.

Our approach presents some limitations. One limit is the dependence on expression data analysis to identify affected pathway, mainly based on the use of changes in gene expression that are largest in size or level. However, some studies [31] show that even small changes in expression levels, which seems not to be of greatest functional significance, can be relevant in terms of phenotypic difference. For example a yeast cell containing a mutation in a gene that confers temperature sensitivity, thus essential for survival at non-permissive temperature, can indeed growth at the normal or permissive temperature. The temperature-sensitive gene in this mutant cell usually has a point mutation that leads to subtle changes in its protein production, but has a high impact in the protein function and thus in the phenotype of the cell. Thus, one possible risk of our approach is to lose significant features from a biological point of view (e.g. significant pathways), even if not from a statistical point of view. Furthermore, in our work, as in common in many studies, there is also a dependency in the data analysis workflow based on the used public datasets. Computational methods that use biological information (as pathways) from prior knowledge could have a bias towards pathways or genes that are better known, since they are more present in the literature and databases [32–34]. Moreover, different pathway databases can give different list of genes of the same pathway leading to affect the results of the computational method. On the other hand, public experimental data could derive by different experimental conditions and/or lack of standardizations of experimental designs. In addition, the details of cell-specific information or clinical data of samples are not always available [32–34]. However, we optimized our computational method based on the reported databases by reducing the bias on data from such databases performing a Monte Carlo cross-validation.

As overall result, our approach have generated promising hypotheses for identifying altered pathways as potential therapeutic targets, either by using synergistic drug combinations and new/approved drugs. Despite the number of drugs is increasing, the specific molecular mechanisms underlying drug combinations with therapeutic effect remain often unclear. We believe that the current efforts based on pathway cross-talk drug strategies will provide key information that are required to decipher molecular mechanism contributing to resistance or inefficacy of drugs.

## Conclusions

Overall, our computational method has identified a set of new potential drug targets that have a large impact on cross-talk inhibition. We consider that further experiments are required to enable the translation of new drug target pathways into therapeutic strategies, however our results show that methods focusing on the Monte Carlo cross-validation and PCI could offer valuable approaches to discover synergistic drugs.

Moreover, we focused our study on BC, but we suggest the application of our approach also to other complex and heterogeneous diseases, in which pathway cross-talk is likely to cooperate important functions.

## Additional files

**Additional file 1.** Shows the network efficiency after inhibition of individual and combined pathway(s) in luminal A BC.

**Additional file 2.** Shows the network efficiency after inhibition of individual and combined pathway(s) in luminal B BC.

**Additional file 3.** Shows the network efficiency after inhibition of individual and combined pathway(s) in HER2 BC.

**Additional file 4.** Shows the network efficiency after inhibition of individual and combined pathway(s) in basal BC.

## Abbreviations

BC: breast cancer
NE: network efficiency
nNE: new network efficiency
DTPN: drug target pathway network
TCGA: The Cancer Genome Atlas
DEG: differentially expressed gene
PCI: pathway cross-talk inhibition
DEA: differential expression analysis
AUC: area under curve
DS: discriminating score
FDA: Food and Drug Administration
